# Ultra-low fluence 532 nm picosecond laser treatment for acne scars: OCT-observed epidermal vacuole-like changes–a case report

**DOI:** 10.3389/fmed.2026.1793087

**Published:** 2026-04-15

**Authors:** Patricia Yu-Chun Peng, Hung-Chia Chang, Hsien-Li Peter Peng

**Affiliations:** 1 Kaohsiung Medical University Hospital, Kaohsiung, Taiwan; 2 P-Skin Professional Clinic, Kaohsiung, Taiwan; 3 Department of Dermatology, Tri-Service General Hospital, National Defense Medical University, Taipei, Taiwan

**Keywords:** acne scars, case report, LIOB, Optical Coherence Tomography (OCT), picosecond 532 nm laser

## Abstract

**Background:**

Treating atrophic acne scars in Asian skin (Fitzpatrick types III–V) is challenging due to the elevated risk of post-inflammatory hyperpigmentation (PIH). While picosecond lasers with fractional handpiece treatment are effective, the optimal balance between fluence, passes, clinical outcome, and downtime remains controversial.

**Case presentation:**

A 28-years-old Asian male with moderate atrophic acne scars was treated with a 532-nm picosecond Nd:YAG laser using a fractional handpiece. An ultra-low fluence of 0.03 J/cm^2^ was applied (8-mm spot size, 4 passes, 2,511 total pulses).

**Results:**

Clinical follow-up demonstrated observable changes in scar contour and skin surface irregularity after a single treatment session, without downtime or PIH. Optical Coherence Tomography (OCT) revealed intraepidermal vacuole-like structures exclusively at the ultra-low setting (0.03 J/cm^2^), while higher fluences (0.05–0.2 J/cm^2^) failed to generate similar discrete OCT findings.

**Conclusion:**

Ultra-low fluence, high-pulse-count 532-nm treatment may be associated with OCT-detectable intraepidermal vacuole-like response not observed at higher fluences. While the biological mechanism remains to be elucidated, these findings highlight the possibility of a non-linear dose–response phenomenon and support further investigation into sub-threshold treatment paradigms in pigment-prone populations.

## 1 Introduction

The management of atrophic acne scars in Asian populations is often limited by the adverse effects of traditional thermal-based treatments. For instance, fractional CO_2_ lasers frequently result in prolonged erythema and a high incidence of post-inflammatory hyperpigmentation (PIH). The advancement of fractional picosecond laser technology has shifted the therapeutic paradigm from photothermal injury to photoacoustic remodeling.

Recent histological evidence suggests that picosecond-domain pulses induce unique tissue reactions: Laser-Induced Optical Breakdown (LIOB) within the epidermis and Laser-Induced Cavitation (LIC) within the dermis (1–5). While fractional 755-nm picosecond lasers typically involve transient erythema resolving within 24 h (6), 1064-nm/532-nm Nd:YAG protocols often utilize high-energy settings associated with 3–7 days of downtime, including petechiae, oozing, and swelling (7–10).

In this report, we describe clinical outcomes following an ultra-low fluence 532-nm picosecond laser protocol combined with real-time OCT imaging. We also present a small retrospective comparison across different fluence settings to explore potential fluence-dependent imaging patterns.

## 2 Materials and methods

### 2.1 Clinical case

A 28-years-old Asian male (Fitzpatrick IV) presented with bilateral atrophic acne scars involving the cheeks. Physical examination revealed mainly rolling and shallow boxcar-type scars distributed across the malar regions, producing mild to moderate surface irregularity. Scattered erythematous macules and occasional inflammatory papules were also noted, with no hypertrophic scars or keloids observed.

**FIGURE 1:**
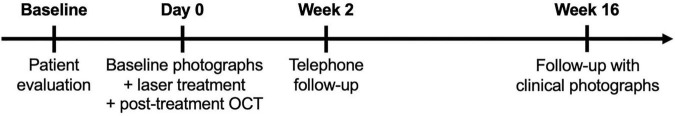
Clinical timeline summarizing the key events of the case.

The patient received a single session of 532-nm fractional picosecond laser treatment (Discovery PicoPlus, Quanta System). The treatment parameters included a fluence of 0.03 J/cm^2^, an 8-mm spot size, and a pulse duration of 375 ps. The procedure consisted of two full-face passes followed by two additional targeted passes over the cheek scars, with a total of 2,511 laser pulses delivered. The overall clinical course and follow-up schedule of the case are summarized in Figure 1.

### 2.2 Retrospective cohort and OCT fluence gradient

To further evaluate the OCT-detected intraepidermal vacuole-like structures observed in the index case, we conducted a retrospective chart review of patients treated at our center using the same 532-nm fractional picosecond handpiece with an 8-mm spot size. The purpose of this analysis was to establish a fluence-gradient comparison model and examine whether the appearance of intraepidermal vacuole-like structures on OCT demonstrated a dose-dependent pattern across varying energy settings.

Three additional patients were identified to serve as a comparative fluence gradient cohort. All patients were Fitzpatrick skin type IV and underwent facial rejuvenation using the same laser platform, with fluence being the only variable parameter. A 35-years-old female received treatment at 0.05 J/cm^2^ specifically for large pore improvement. A 57-years-old female was treated at 0.07 J/cm^2^ for facial rejuvenation. A 47-years-old female underwent treatment at 0.20 J/cm^2^ for facial rejuvenation. Treatment technique, spot size, and device configuration were otherwise consistent across all cases.

To evaluate the reproducibility of the low-fluence response, five patients, including the index case, treated at 0.03 J/cm^2^ were reviewed to assess the consistency of intraepidermal vacuole formation on OCT imaging. Cross-case image analysis was performed to determine whether vacuole formation occurred reliably at this setting, thereby supporting the hypothesis of a definable biological activation threshold rather than an isolated event. This fluence-stratified comparison enabled preliminary characterization of a potential non-linear imaging response across energy levels.

### 2.3 Digital photography and OCT imaging

Standardized 2D digital photographs were captured at baseline and follow-up using a Nikon D610 camera. OCT imaging was utilized immediately post-treatment to assess tissue reactions.

### 2.4 Ethical considerations

All clinical procedures and imaging were performed in accordance with the Declaration of Helsinki. While this specific retrospective review did not utilize a formal Institutional Review Board (IRB) approval number due to its basis in routine clinical practice, all patients provided written informed consent for their de-identified clinical and imaging data to be used for research and publication.

### 2.5 Literature review

A systematic minireview of clinical literature (2016–2025) was conducted, focusing on picosecond laser parameters, efficacy, and downtime for acne scars in Asian skin. Thirteen key sources were analyzed to provide a benchmark for the current case (Table 1).

**TABLE 1:** Clinical summary of picosecond laser treatment for atrophic acne scars.

References	Wavelength (nm)	Pulse duration	Fluence/pulse energy	Passes/ sessions	Clinical endpoint	Downtime	Improvement/ results
Tanghetti (1)	755	ps	0.25–0.71 J/cm^2^	1–3 passes (*Ex vivo*)	LIOB (histology)	N/A	LIOB size/density correlate with melanin.
Bernstein et al. (7)	1064/532	450/375 ps	1064: 0.83–1.1 J/cm^2^ 532: 0.13–0.18 J/cm^2^	4 sessions (2 passes)	Petechiae, erythema	4–5 days	75% identification of improvement.
Tanghetti and Jennings (2)	755/1064/532	ps	532: 0.2–0.28 mJ 1064: 1.3–2.1 mJ	1 session (3 passes)	Petechiae (Nd:YAG)	24 hrs	Nd:YAG creates more purpura than 755 nm.
Dai et al. (3)	1064	450 ps	0.7–1.0 J/cm^2^	3 sessions (2 passes)	Pinpoint bleeding	4 days (median)	Mean clinical improvement of 3.25/10.
Huang et al. (11)	755	750 ps	0.71 J/cm^2^	4.28 sessions (mean)	Texture improvement	<24 hrs	4.7% PIH rate in 42 Asian patients.
Huang et al. (6)	755	ps	0.71 J/cm^2^	4–6 sessions	Longterm-stability	N/A	Durable results sustained up to 3 years.
Hwang et al. (14)	1064	ps	1.2 J/cm^2^	Serial OCT	LIOB (OCT)	N/A	Observed dynamic serial changes of LIOB.
Yeh et al. (4)	1064/532	450/375 ps	High: 1064(2.3)/532(0.3) J/cm^2^ Low: 1064(1.9)/532(0.2) J/cm^2^	*Ex vivo* histology	LIOB vs. LIC	N/A	High energy induces LIOB; Low induces LIC.
Yang et al. (8)	1064/532	450/375 ps	532(F): 0.3–0.5 J/cm2	3 sessions (3-step)	Mild petechiae	3–7 days	Average ECCA reduction of 28.3%.
			1064(F): 1.5–1.9 J/cm^2^				
			1064(S): 2.1–2.5 J/cm^2^				
Shi et al. (9)	1064/532	450 ps	1064: 1.7–1.9 J/cm^2^	3 sessions (8 passes)	Erythema, petechiae	3–5 days	Comparable efficacy to 1540 nm Er:glass.
			532: 0.2–0.22 J/cm^2^				
O’Connor et al. (5)	1064/532	450 ps	1064: 6.0(H)/10.5(L) J/cm^2^	1 session (*in vivo*)	LIOB and LIC (histology)	<2 weeks	Epidermal LIOB and dermal LIC identified for both.
			532: 3.2(H)/1.0/0.4(L) J/cm^2^				
Lee et al. (12)	1064	450 ps	0.3 vs. 1.0 J/cm^2^	3 sessions	Petechiae/pain	2.3–3.4 days	Both fluences effective; HF has more downtime.
Shimojo et al. (15)	532/1064	ps	532: 0.95 J/cm^2^ 1064: 6.50 J/cm^2^	Basic science	Melanosome disruption	N/A	532 nm threshold is significantly lower.
Jia et al. (10)	1064/532	ps	1064F: 0.6–0.8 J/cm^2^ 532F: 0.16–0.2 mJ	1–10 sessions	Pinpoint bleeding	5–7 days	GASIS 1.05; improvement correlated with sessions.
Current case	532	375 ps	0.03 J/cm^2^	1 session (4 passes)	Mild erythema	None	Intraepidermal vacuole-like structures (+) observed only at 0.03 J/cm^2^ via OCT.

## 3 Results

### 3.1 Clinical outcomes

Immediately after treatment, mild transient erythema was observed without petechiae or epidermal crusting. The erythema resolved within 30 min.

At a 2-weeks telephone follow-up, the patient reported no adverse effects and noted subtle early improvement in skin texture.

**FIGURE 2:**
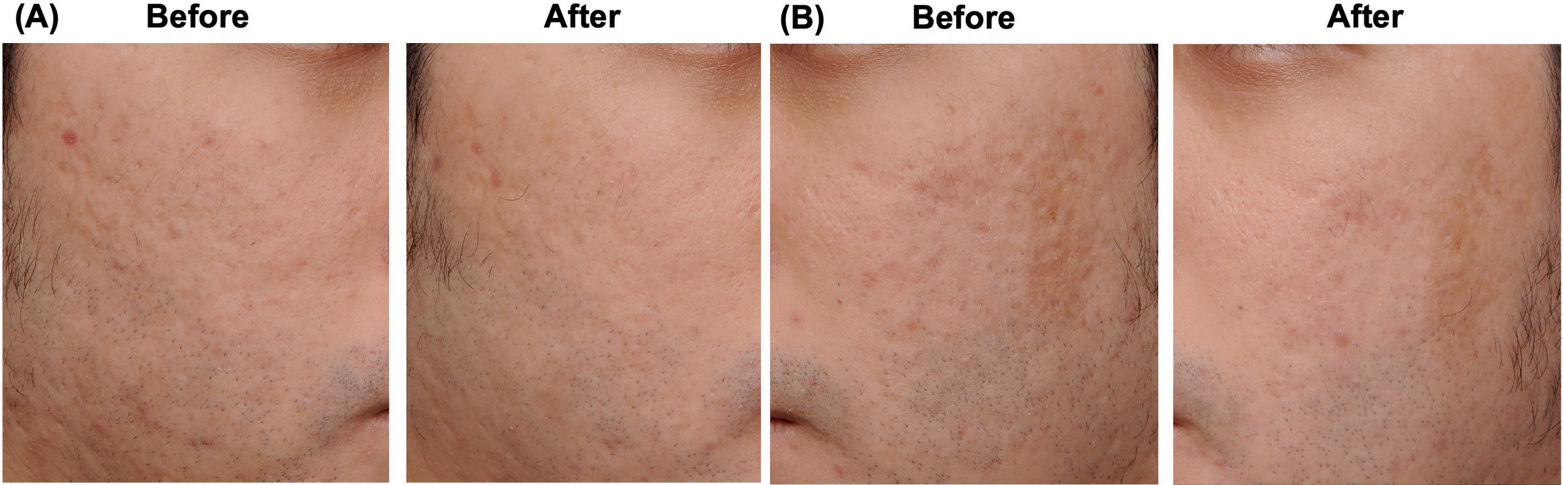
Clinical photographs of bilateral cheek acne scars before and 16 weeks after a single session of ultra-low fluence 532-nm Nd:YAG picosecond laser treatment (0.03 J/cm^2^). **(A)** Right cheek. **(B)** Left cheek.

At the 16-weeks follow-up, changes in scar contour and skin surface irregularity were clinically observed, with the treated areas appearing smoother compared with baseline (Figures 2A, B). These observations were based on physician clinical assessment and patient-reported perception rather than objective quantitative scar scoring. No post-inflammatory hyperpigmentation or other complications were documented. The patient reported satisfaction with the treatment outcome and perceived improvement in skin texture.

### 3.2 Outcomes of comparison cohort

The retrospective comparison cohort demonstrated a fluence-associated increase in treatment-related discomfort and downtime.

At 0.05 J/cm^2^, the subject reported no pain during treatment and experienced mild post-laser erythema lasting 1–2 days. Decreased skin surface irregularity was observed by the evaluating physician at 2 weeks post-treatment.

At 0.07 J/cm^2^, the subject reported mild procedural discomfort (VAS 2–3) and exhibited mild erythema persisting for 2–3 days. Similar clinical changes in skin texture were observed by the evaluating physician at 2 weeks post-treatment.

At 0.20 J/cm^2^, the subject reported moderate discomfort (VAS 5–6) and developed moderate erythema lasting 3–5 days. Similar clinical changes in skin texture were observed at 2 weeks post-treatment.

### 3.3 OCT imaging results

**FIGURE 3:**
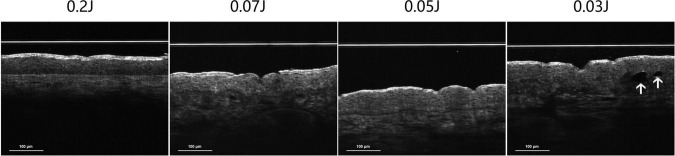
Optical Coherence Tomography (OCT) obtained immediately after 532-nm fractional picosecond laser treatment at different fluence settings (0.03, 0.05, 0.07, and 0.2 J/cm^2^). Intraepidermal vacuole-like structures (white arrows) are observed at the ultra-low fluence of 0.03 J/cm^2^ (index case), whereas similar discrete structures are not apparent at higher fluences.

Optical Coherence Tomography assessment immediately after 532-nm treatment revealed a paradoxical fluence-dependent imaging pattern. Intraepidermal vacuole-like structures were observed exclusively at the ultra-low fluence of 0.03 J/cm^2^. In contrast, higher fluences (0.05–0.20 J/cm^2^), despite producing more pronounced clinical endpoints such as increased discomfort and erythema, did not demonstrate discrete intraepidermal vacuole-like structures on OCT (Figure 3). These findings suggest a non-linear relationship between fluence and OCT-detectable epidermal microstructural alterations.

## 4 Discussion

### 4.1 Advances in picosecond technology and LIOB/LIC formation

Fractional picosecond handpieces have shifted tissue interaction from predominantly photothermal injury toward photomechanical effects. Focused laser energy delivered to the epidermal focal zone may generate micro-structural tissue responses that have been described in previous histologic studies as laser-induced optical breakdown (LIOB) in the epidermis and Laser-Induced Cavitation (LIC) in the dermis. These micro-vacuoles initiate a regenerative cascade, increasing collagen, elastin, and mucin, thereby improving atrophic scars (11, 12).

### 4.2 Non-invasive observation via OCT: the LIOB paradox

In the present study, OCT was utilized to non-invasively assess immediate post-treatment tissue architecture. Intraepidermal vacuole-like structures were observed exclusively at the ultra-low fluence of 0.03 J/cm^2^. In contrast, higher fluences (0.05–0.20 J/cm^2^), despite producing greater discomfort and longer erythema duration, did not demonstrate similar discrete optical voids.

This unexpected pattern represents a paradoxical, non-linear fluence-dependent imaging response. Conventionally, higher energy delivery is assumed to enhance photomechanical effects; however, our findings suggest that detectable microstructural alterations may occur within a narrow fluence window. The association of these vacuole-like structures with Laser-Induced Optical Breakdown (LIOB) remains speculative, as definitive histological confirmation was not performed in this cohort.

The underlying physical basis for the absence of clearly visible vacuoles on OCT imaging at higher fluences remains uncertain. Several mechanistic hypotheses may account for this paradoxical finding and warrant prospective investigation.

First, a thermal masking effect may occur at higher fluences, whereby increased thermal diffusion and bulk tissue heating obscure the sharply demarcated boundaries of mechanically induced vacuoles. Rather than discrete optical voids, surrounding coagulative or sublethal thermal changes may reduce refractive index contrast, thereby diminishing OCT detectability.

Second, the optical precision of the microlens array (MLA) may be optimized only within a narrow low-fluence window. It is conceivable that precise plasma formation and confined laser-induced optical breakdown (LIOB) occur most efficiently at specific energy outputs, whereas higher fluences may induce broader energy dispersion or overlapping microthermal zones that reduce focal confinement.

Third, emerging evidence suggests that the melanosome disruption threshold at 532 nm may be lower than previously assumed. If melanosomes serve as primary absorptive targets for plasma initiation, sub-threshold fluences may still be sufficient to induce localized photomechanical effects without generating overt thermal signatures, thereby supporting the possibility of LIOB formation at unexpectedly low energy settings.

Together, these hypotheses suggest that the observed phenomenon may reflect a complex interaction between optical focusing dynamics, tissue absorption characteristics, and thermomechanical balance rather than a simple linear dose–response relationship.

### 4.3 Treatment protocols and synergy

Histological studies have demonstrated that both 1064-nm and 532-nm picosecond laser wavelengths can induce epidermal laser-induced optical breakdown (LIOB) as well as dermal laser-induced cavitation (LIC) (4, 5). These phenomena appear to be influenced by wavelength, fluence, focal configuration, and tissue optical properties.

In our previous studies, higher-fluence 1064-nm settings have been associated with dermal cavitation patterns consistent with LIC, whereas relatively lower fluences have been associated with more confined epidermal photomechanical changes described as LIOB. Similarly, at 532 nm, variations in fluence have been reported to produce differing tissue interaction profiles.

While the present study does not provide histologic confirmation of LIOB or LIC, the observed non-linear OCT imaging response raises the possibility that clinically relevant photomechanical effects may occur within specific fluence ranges rather than increasing proportionally with energy delivery.

Some investigators have advocated higher-energy settings to enhance LIOB formation and improve clinical outcomes (3), whereas other split-face studies have demonstrated comparable efficacy between lower- and higher-fluence treatments (12), underscoring ongoing uncertainty regarding optimal parameter selection in picosecond laser therapy.

### 4.4 New clinical strategy for Asian skin

Our group has previously reported that 755-nm picosecond lasers achieve significant atrophic acne scar improvement in Asian patients, with a low incidence of post-inflammatory hyperpigmentation (4.7%) and evidence of long-term durability of clinical outcomes (6, 11). In contrast, high-fluence 1064/532-nm protocols are frequently associated with pinpoint bleeding and 5–7 days of post-procedural downtime (7–10).

In the present case, the use of an ultralow energy setting of 0.03 J/cm^2^ resulted in favorable clinical efficacy without petechiae, epidermal crusting, post-inflammatory hyperpigmentation, or treatment-related downtime. Notably, intraepidermal vacuole-like structures were reproducibly observed on OCT at this ultra-low setting, whereas higher fluences (0.05–0.20 J/cm^2^) were associated with progressively increased discomfort (VAS 0–6) and erythema duration (1–5 days) but did not demonstrate similar discrete OCT findings.

These observations suggest that clinically meaningful tissue responses may occur within a narrow “sub-threshold” fluence window. While the relationship between these OCT findings and classical LIOB remains unconfirmed, the data raise the possibility that effective remodeling in pigment-prone Asian skin may not require high-energy endpoints traditionally associated with greater downtime.

## 5 Limitations

While this study provides preliminary clinical and imaging observations suggesting a paradoxical fluence-dependent OCT response, several important limitations must be acknowledged.

First, the study design inherently limits generalizability. As a single index case supported by a small retrospective review of five patients, the findings may not be broadly applicable to the wider Asian population or to other Fitzpatrick skin types. The exploratory nature of this analysis necessitates cautious interpretation.

Second, a key methodological limitation is that the fluence comparison was performed across different individuals rather than within the same subject. Although patients were matched for Fitzpatrick skin type IV and treated using identical device parameters aside from fluence, inter-individual variation in skin characteristics–including epidermal thickness, dermal hydration, age-related structural differences, and scar morphology–may have influenced OCT imaging findings. Consequently, these observations cannot exclude confounding effects arising from biological differences between subjects. Future investigations using intra-individual controlled designs, such as split-face or split-area studies evaluating multiple fluence settings within the same patient, will be necessary to determine whether the observed OCT patterns represent a reproducible fluence-dependent phenomenon.

Third, the lack of histological validation means that the observed vacuole-like structures on OCT should be interpreted with caution and cannot be considered definitive evidence of LIOB. Although Optical Coherence Tomography is a validated, non-invasive modality for assessing tissue architecture and has been used to visualize intraepidermal vacuole-like structures in both animal (13) and human models (14), the vacuoles observed in this study were not confirmed by biopsy. Without direct histopathologic correlation, there remains a risk of overinterpreting OCT findings as definitive evidence of laser-induced optical breakdown (LIOB). Imaging-based inference, while suggestive, cannot substitute for histological validation.

Additionally, the retrospective nature of the comparison cohort introduces inherent bias. The fluence gradient data were derived from routine clinical practice rather than a pre-designed prospective protocol. Formal Institutional Review Board (IRB) approval was not obtained at the time of treatment, as these interventions were performed as part of standard care; however, all patients provided written informed consent for the use of de-identified clinical and imaging data. Nonetheless, retrospective chart review limits control over confounding variables and outcome standardization.

Furthermore, the comparator patients were treated for heterogeneous clinical indications, including facial rejuvenation and large pore improvement, rather than specifically for atrophic acne scars. While immediate procedural endpoints such as pain and erythema were documented, long-term clinical outcomes are not directly comparable to those of the index case, limiting interpretation of durability and efficacy within a scar-specific framework.

Finally, the mechanistic explanation for the absence of visible vacuoles on OCT at higher fluences remains unresolved. Proposed hypotheses–including thermal masking effects and fluence-dependent microlens array focal precision–remain speculative and lack direct experimental validation. The physical interaction between energy density, optical focusing dynamics, and tissue response requires controlled laboratory investigation to substantiate these theories.

Further research through prospective randomized controlled trials, larger cohort studies, intra-subject dose–response designs, and longitudinal follow-up is essential to validate the safety, reproducibility, and durability of this ultra-low energy, sub-threshold paradigm. Only through systematic investigation can the proposed LIOB paradox be definitively characterized and translated into evidence-based clinical practice.

## 6 Conclusion

A single session of ultra-low energy (0.03 J/cm^2^) 532-nm fractional picosecond laser treatment was associated with clinically observable changes in atrophic acne scars in this case without significant downtime or post-inflammatory hyperpigmentation.

In this small observational series, intraepidermal vacuole-like structures observed on OCT were detected at the ultra-low fluence setting but were absent at higher fluence levels (0.05–0.2 J/cm^2^). The biological significance of these OCT findings remains uncertain, and their relationship to laser-induced optical breakdown (LIOB) cannot be determined without histologic confirmation.

These preliminary findings suggest the possibility of a non-linear fluence-response relationship in picosecond-laser induced tissue changes. Further controlled studies with larger cohorts and histologic correlation are required to clarify the underlying mechanisms and clinical implications of this ultra-low fluence treatment approach.

## Data Availability

The raw data supporting the conclusions of this article will be made available by the authors, without undue reservation.
